# Supplementary material for: Graphene-Based Electrodes in a Vanadium Redox Flow Battery Produced by Rapid Low-Pressure Combined Gas Plasma Treatments
Source: Chem Mater. 2021 May 26;33(11):4106–21. doi: 10.1021/acs.chemmater.1c00763 (PMC8274967; doi:10.1021/acs.chemmater.1c00763)
Supplement: Supplementary file 2 — cm1c00763_si_002.pdf [file cm1c00763_si_002.pdf]

Supporting Information

Graphene-based electrodes in a vanadium redox flow battery produced by rapid low-pressure combined gas plasma treatments

Sebastiano Bellani,[*,‡,#,§] *Leyla Najafi,*[‡, #,§] *Mirko Prato,*[‼] *Reinier Oropesa-Nuñez,*[#,ǁ] *Beatriz Martín-García,*[§,Γ] *, Luca Gagliani,*[§] *Elisa Mantero,*[#,§] *Luigi Marasco,*[§] *Gabriele Bianca,*[§,ǂ] *Marilena I. Zappia,*[#,↧] *Cansunur Demirci,*[ǂ,ˀ] *Silvia Olivotto,*[˥] *Giacomo Mariucci,*[□] *Vittorio Pellegrini,*[#,§] *Massimo Schiavetti*[⁉] *and Francesco Bonaccorso*[*,#,§]

[#]BeDimensional S.p.a., Via Lungotorrente secca 3D, 16163 Genova, Italy

[§]Graphene Labs, Istituto Italiano di Tecnologia, via Morego 30, 16163 Genova, Italy

[ǁ]Department of Materials Science and Engineering, Uppsala University, Box 534, 75121 Uppsala, Sweden

[‼]Materials Characterization Facility, Istituto Italiano di Tecnologia, via Morego 30, 16163 Genova, Italy

[ǂ]Dipartimento di Chimica e Chimica Industriale, Università degli Studi di Genova, via Dodecaneso 31, 16146 Genoa, Italy

[↧]Department of Physics, University of Calabria, via P. Bucci cubo 31/C, 87036 Rende, Cosenza, Italy

[ˀ]NanoChemistry, Istituto Italiano di Tecnologia, via Morego 30, 16163 Genova, Italy

[˥]Wind Technology Innovation, Enel Global Power Generation

[⁉]Thermal & Industry 4.0 Innovation, Enel Global Power Generation

[□]Storage and New Business Design, Engineering & Construction, Enel Green Power S.p.A.

[Γ]CIC nanoGUNE, 20018 Donostia-San Sebastian, Basque, Spain

Methods

Graphene production. Single-/few-layer graphene flakes were produced through wet-jet milling exfoliation (WJM) of graphite.[1,2] Experimentally, a mixture including 20 L of N-Methyl-2-pyrrolidone (> 97%, Sigma Aldrich) and 200 g of graphite flakes (+100 mesh, Sigma Aldrich) are mixed in a container by a mechanical stirrer (Eurostar digital Ika-Werke). Nextly, the mixture is pushed by a hydraulic piston into a processor consisting of five sets of different perforated and interconnected disks by applying a 250 MPa pressure. Two jet streams originate at the second disk, which is made of two holes with a diameter of 1 mm in diameter. Subsequently, the jet streams collide between the second and the third disks, which consists of a nozzle with diameter of 0.3 mm. During the passage of the sample through the nozzle, the turbulence of the solvent originates the shear force causing the exfoliation of the graphite. The theory of the exfoliation mechanism has been described in ref. [1]. The as-produced dispersion is cooled down by a chiller and then collected in another container.

Then, the sample is re-processed by WJM apparatus two times, passing consecutively through nozzles with diameters of 0.15 mm and 0.1 mm.

Characterization of graphene. Transmission electron microscopy (TEM) images were taken with a JEM 1011 (JEOL) transmission electron microscope, operating at 100 kV. Morphological and statistical analysis were carried out by using ImageJ software (NIH) and OriginPro 9.1 software (OriginLab), respectively. The samples were prepared by drop casting the 1:50 diluted graphene dispersions onto ultrathin C-film on holey carbon 400 mesh Cu grids (Ted Pella Inc.). The grids were stored under vacuum at room temperature overnight to remove the solvent residues. The AFM measurements were carried out using an Innova AFM (Bruker, Santa Barbara, CA) in intermittent contact mode, TESPA cantilevers (Bruker, Santa Barbara, CA) with a nominal tip diameter of 16 nm and a drive frequency of ~320 kHz. The AFM images were acquired over an area of 5×5 μm^2 (512×512 data points) by keeping the working set point above 70% of the free oscillation amplitude. The scan rate for the acquisition of images was 0.7 Hz. The height profiles were processed using the Gwyddion (version 2.43) software and the data were analysed with OriginPro 9.1 software. Statistical analysis was carried out by means of Origin 9.1 software on multiple AFM images. The samples were prepared by drop-casting the 1:120 diluted graphene dispersion onto freshly-cleaved mica sheets (G250-1, Agar Scientific Ltd., Essex, U.K.) and dried under vacuum at room temperature overnight to remove the solvent residues. Raman spectroscopy measurements were carried out using a Renishaw micro-Raman inVia 1000 with a 50× objective (numerical aperture of 0.75), an excitation wavelength of 514.5 nm and an incident power on the samples of 5 mW. The samples were prepared by drop casting the 1:30 diluted graphene dispersion onto a Si wafer covered with 300 nm thermally grown SiO_2 (LDB Technologies Ltd.). The bulk graphite was analysed in its powder form. For each sample, 50 spectra were collected. OriginPro 9.1 software was used to perform the deconvolution and statistics. X-ray photoelectron spectroscopy (XPS) analysis was carried out using a Kratos Axis Ultra^{DLD} spectrometer. The XPS spectra were acquired using a monochromatic Al K_{α} source operating at 20 mA and 15 kV. The analysis was carried out over an area of 300 µm × 700 µm. High-resolution spectra of C 1s, N1s and O 1s regions were collected at pass energy of 10 eV and energy step of 0.1 eV. Energy calibration was performed setting the C-C peak in C 1s spectra at 284.8 eV. Data analysis was carried out with CasaXPS software (version 2.3.19).

Scanning electron microscopy analysis of the pristine graphite felt

Figure S1 reports a scanning electron microscopy (SEM) image of the pristine graphite felt (GF), which consists of a bundle of graphitic fibres.

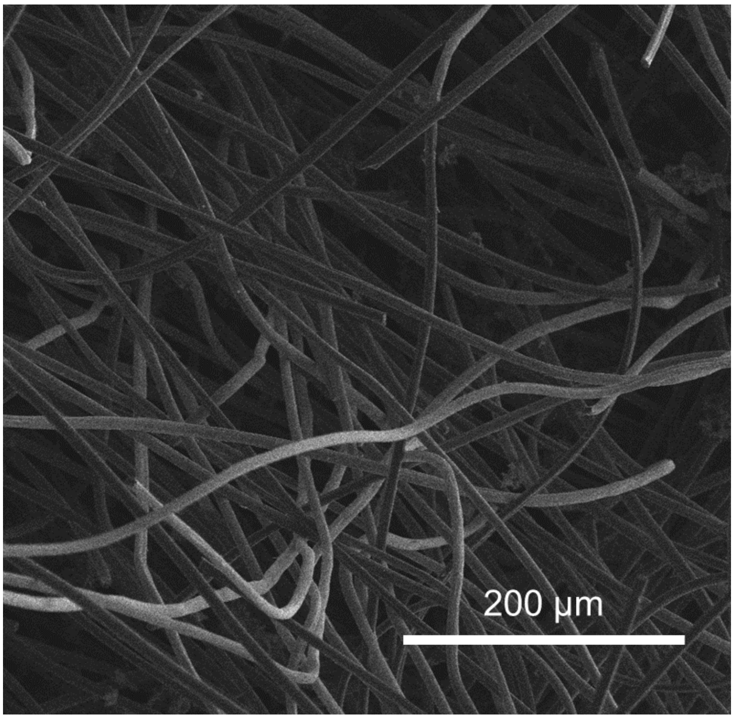

Figure S1. SEM images of the pristine GF.

X-ray photoelectron spectroscopy of the pristine and plasma treated GFs

Figure S2 shows the wide scan XPS spectra of the investigated electrodes. **Figures S3-S5** report the high-resolution XPS spectra in the region of C 1s, O 1s and N 1s for the investigated electrodes. The quantitative analysis of the elemental composition, the O and N functionalities distributions is reported in the main text (**Figure 2**)

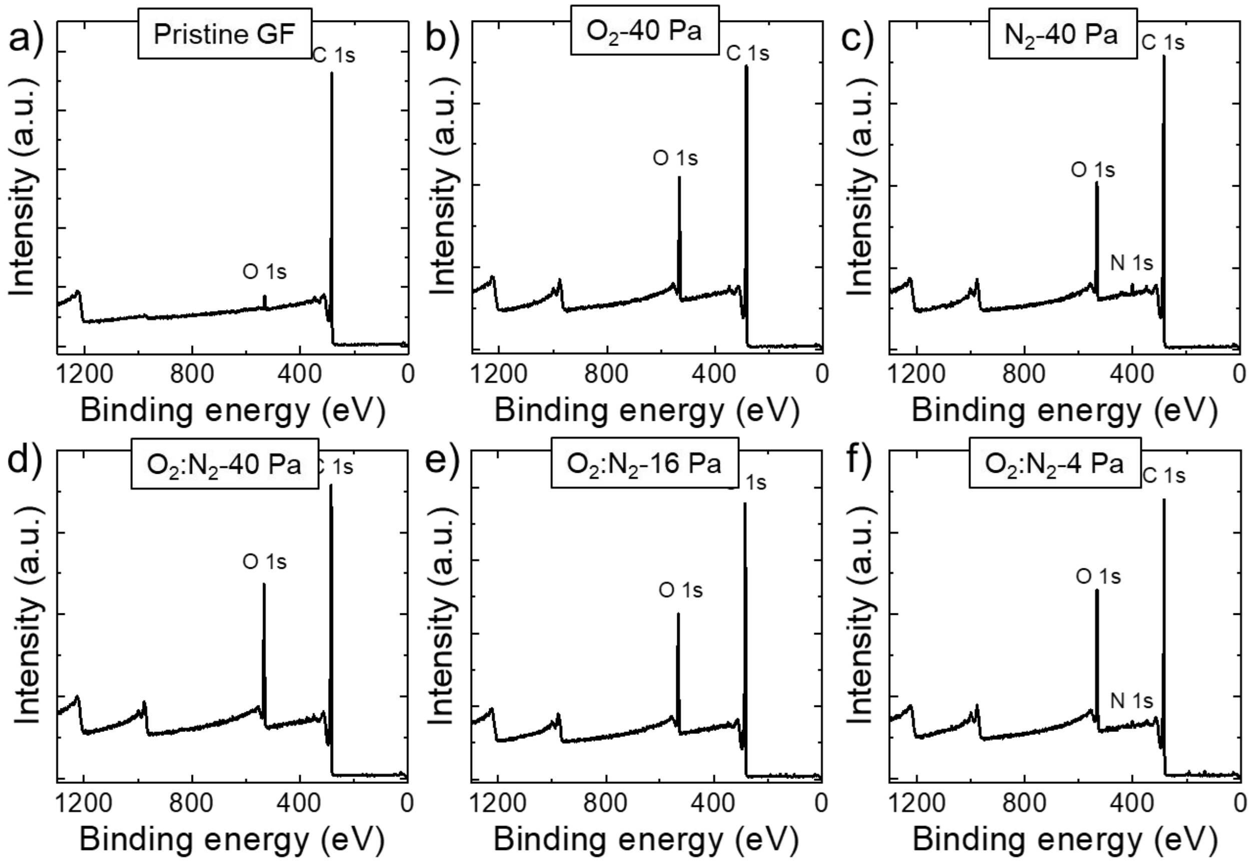

Figure S2. Wide scan XPS spectra of: a) pristine GF, b) O_2-40 Pa, c) N_2-40 Pa, O_2:N_2-40 Pa; e) O_2:N_2-16 Pa and f) O_2:N_2-4 Pa

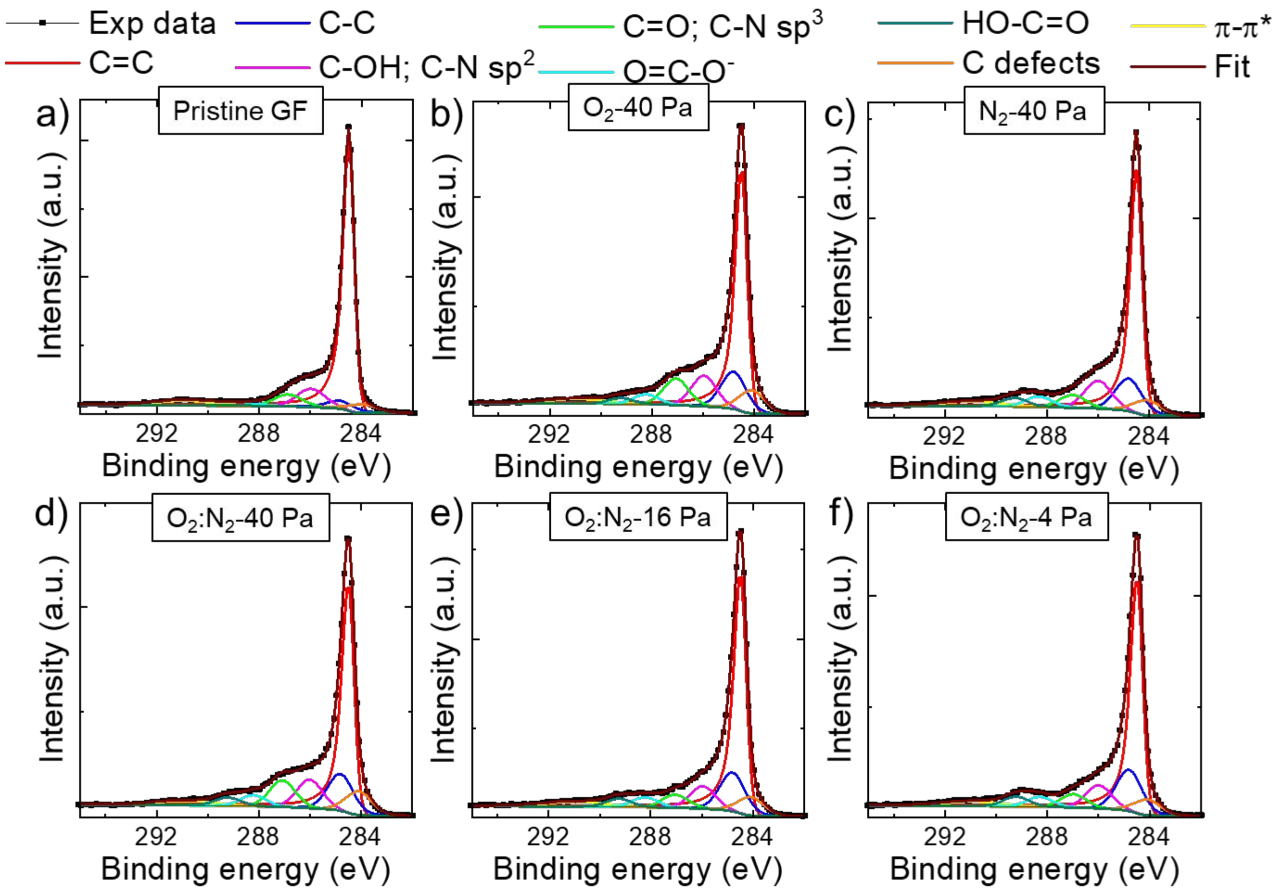

Figure S3. C 1s XPS spectra of: a) pristine GF, b) O_2-40 Pa, c) N_2-40 Pa, O_2:N_2-40 Pa; e) O_2:N_2-16 Pa and f) O_2:N_2-4 Pa. The deconvolutions of the spectra are also shown to evidence the bands attributed to the different oxidation states of C.

The deconvolution of the C 1s spectra reveal the presence of several C environments. **Table S1** summarizes the positions and assignations for each of the observed components, in agreement with literature reports.[3,4,5,6,7,8,9]

Table S1. Positions and assignations of the components observed in the XRD C 1s spectra.

	Binding Energy (eV); Error: ±0.2 eV	Chemical assignation
1	284.5	C=C
2	290.9	π-π*
3	284.8	C-C
4	284.0	C defects
5	286.0	C-OH; C-N sp^2
6	287.0	C=O, C-N sp^3
7	288.2	$O=C-O^-$
8	289.3	O=C-OH

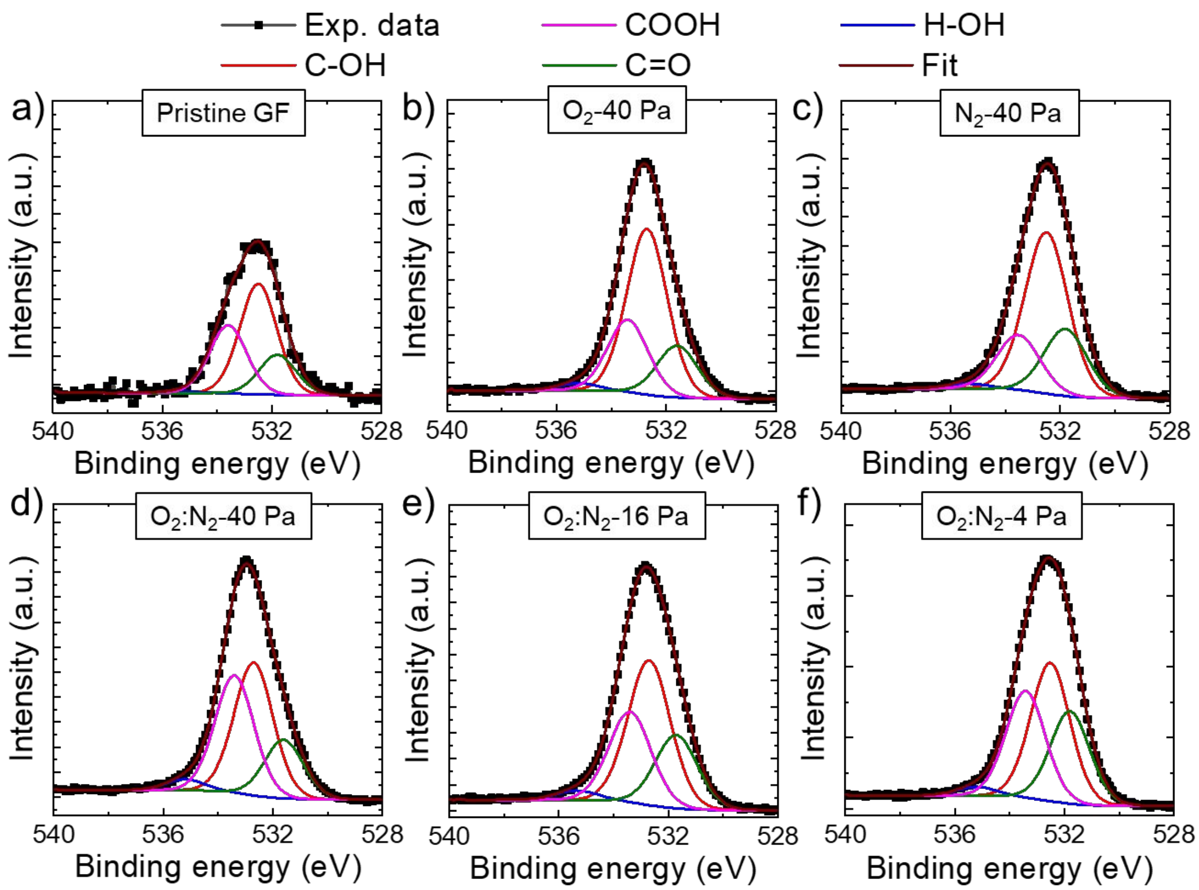

Figure S4. O 1s spectra of: a) pristine GF, b) O_2-40 Pa, c) N_2-40 Pa, O_2:N_2-40 Pa; e) O_2:N_2-16 Pa and f) O_2:N_2-4 Pa. The deconvolutions of the spectra are also shown to evidence the bands attributed to the different O functionalities.

The decomposition of the O 1s spectra and the assignation of the components were performed in accordance with literature.[9,10] In particular, we assigned the peak at (531.7±0.2) eV to C=O groups, the one at (532.6±0.2) eV to C-OH groups, the one at (533.4±0.2) eV to O=C-OH groups, and the one at (535.3±0.2) eV to adsorbed H_2O molecules.

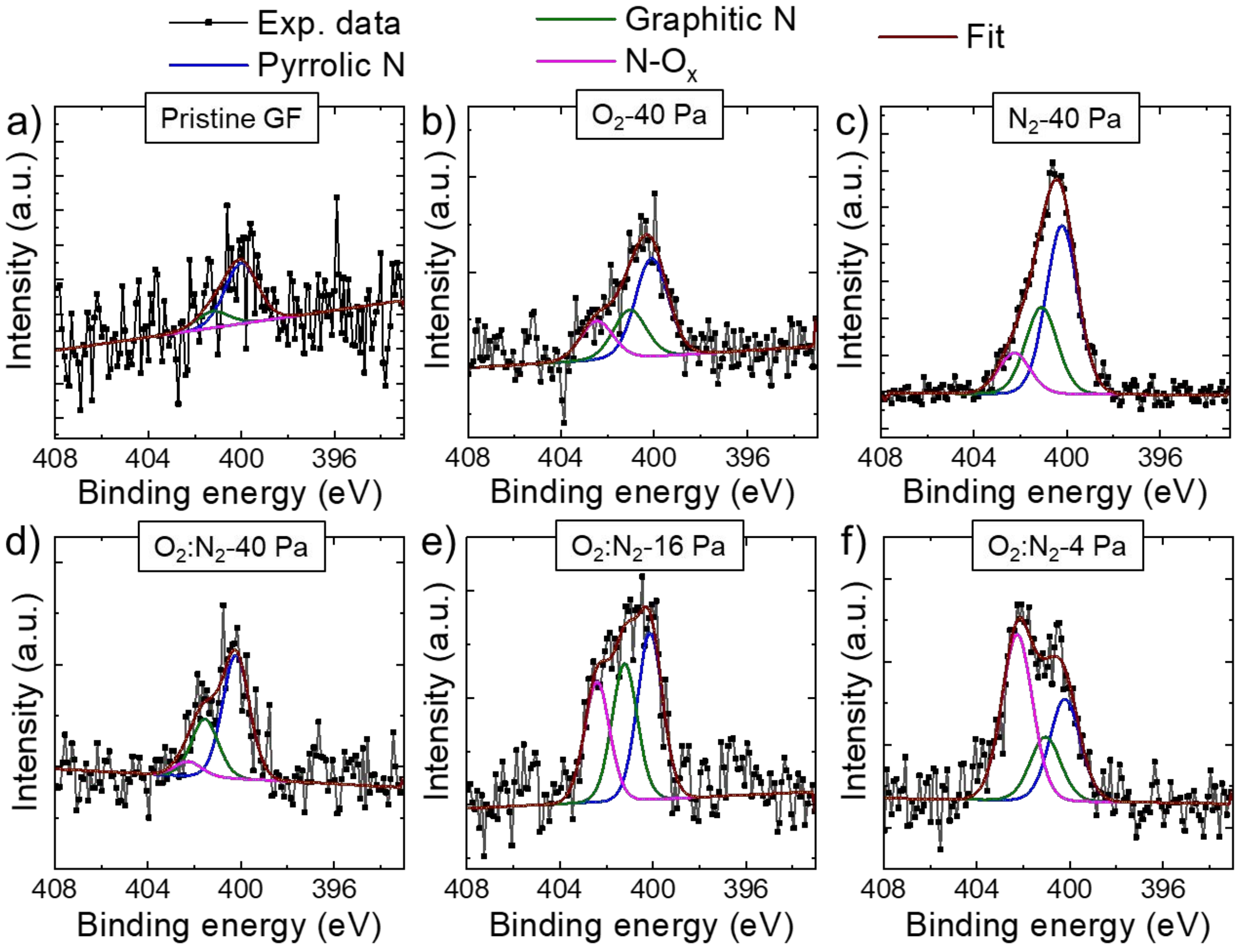

Figure S5. N 1s spectra of: a) pristine GF, b) O_2-40 Pa, c) N_2-40 Pa, O_2:N_2-40 Pa; e) O_2:N_2-16 Pa and f) O_2:N_2-4 Pa. The deconvolutions of the spectra are also shown to evidence the bands attributed to the different N functionalities.

The decomposition of the N 1s spectra and the assignation of the components were performed in accordance with literature. [10] In particular, pyrrolic N was found at (400.1±0.2) eV, graphitic N at (401.2±0.2) eV and N-O_x groups at (402.4±0.2) eV.

Supplementary cyclic voltammetry analysis for the pristine and plasma-treated GFs

Figures S6a,b show the anodic and cathodic current density peaks (I_{pa} and I_{pc}, respectively) as a function of the square root of the potential scan rate for the investigated electrodes. The linear behaviour of the curves indicates that the redox reactions are limited by the transport of the reactants towards the electrode surface, in agreement with the Randles-Sevick equation.

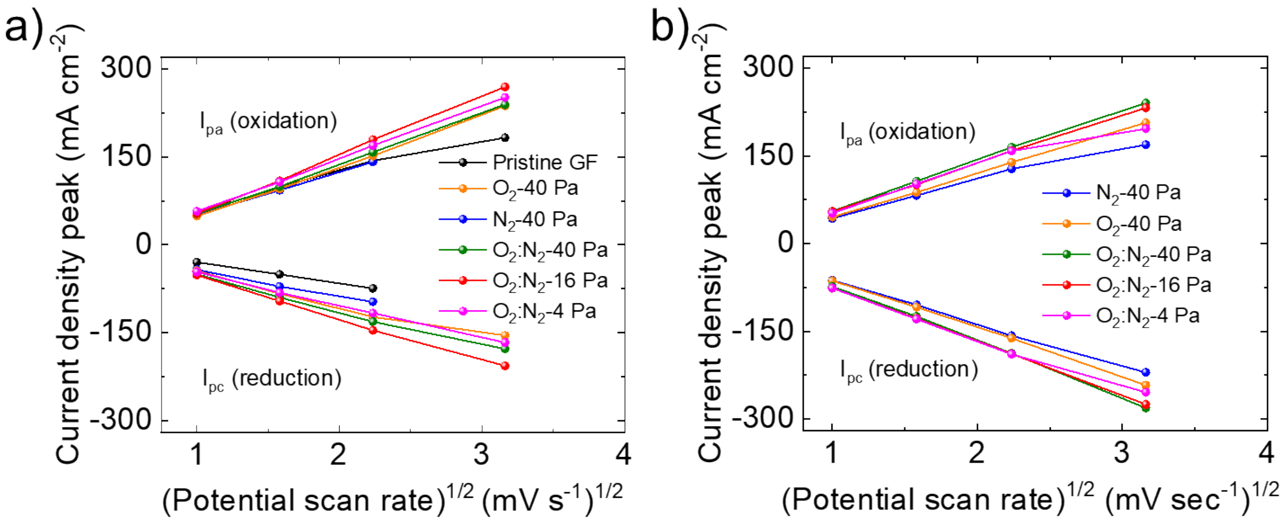

Figure S6. a,b) I_{pa} and I_{pc} as a function of the square root of the potential scan rate measured for the investigated electrode for the VO^{2+}/VO_2^+ and V^{2+}/V^{3+} redox reactions.

Polarization curves without iR-correction of the VRFBs based on pristine and plasma-treated GFs

Figure S7 shows the polarization curves without iR correction (raw data) measured for the VRFBs based on pristine and plasma-treated GFs. The comparison between these data and iR-corrected ones (**Figure 4a**) shows that the Nafion 115 resistance significantly contributes to the overall polarization at current densities higher than 50 mA cm^{-2}.

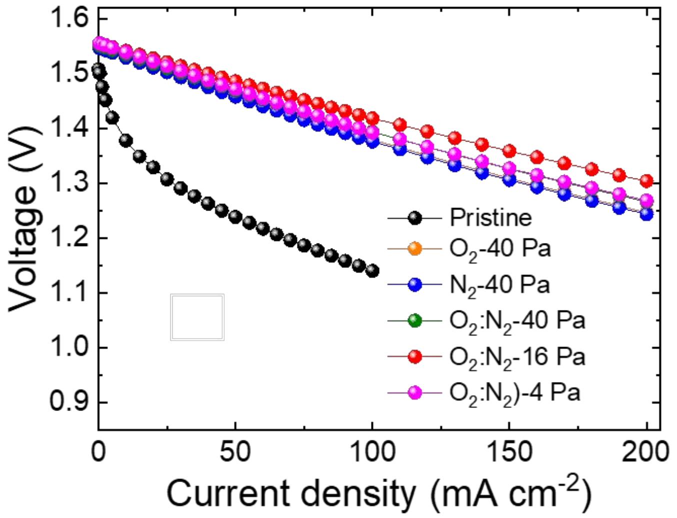

Figure S7. Polarization curves without iR-correction (raw data) measured for the VRFBs using pristine or plasma-treated GFs and Nafion 115 as the proton-exchange membrane.

Polarization curves of the VRFBs based on GFs treated by sequential gas plasmas

Figure S8 shows the iR-corrected polarization curves of the VRFBs based on GFs treated by sequential plasmas with two different gases (*i.e.*, O_2+N_2-16 Pa and N_2+O_2-16 Pa) in comparison to the curve measured for the VRFBs using GFs treated with a single combined gas plasma (*i.e.*, O_2:N_2-16 Pa). Clearly, the combination of O_2 and N_2 gases during the same plasma process lead to inferior kinetic losses in comparison to the use of a sequential gas plasma with different gases. As explained in the text (see **Figure 1,2**), the multiple gas plasma species leads to synergistic effects in modifying the morphological and chemical properties of the GF surfaces during the plasma treatment.

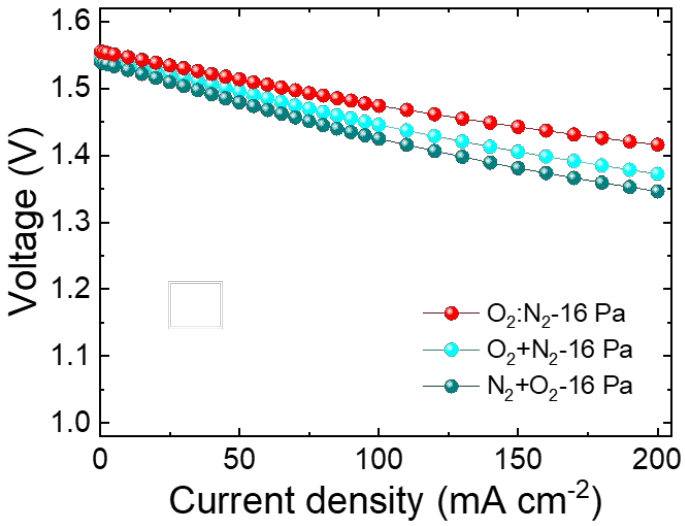

Figure S8. iR-corrected polarization curves measured for the VRFBs using O_2:N_2-16 Pa, or O_2+N_2-16 Pa or N_2+O_2-16 Pa electrodes and Nafion 115 as the proton-exchange membrane.

Efficiency metrics of the VRFBs using Nafion 115

Table S1. Summary of the efficiency metrics of the investigated VRFBs using Nafion 115. The values have been extrapolated from the galvanostatic CD measurements at various current densities and correspond to the second CD cycle.

VRFB	Current density (mA cm^{-2})	CE (%)	VE (%)	EE (%)
Pristine GF	25	97.6	78.7	76.8
	50	98.1	73.2	71.8
	75	98.4	68.5	67.4
	100	98.5	63.9	62.9
O_2-40 Pa	25	97.5	95.0	92.6
	50	98.0	90.4	88.6
	75	98.3	85.7	84.3
	100	98.5	81.1	79.9
N_2-40 Pa	25	97.0	95.4	92.5
	50	97.3	89.8	87.4
	75	97.9	84.5	82.7
	100	98.0	79.9	78.3
O_2:N_2-40 Pa	25	97.0	95.7	92.8
	50	97.4	91.8	89.5
	75	97.6	87.6	85.5
	100	97.8	83.7	81.8
O_2+N_2-40 Pa	25	97.6	91.7	89.5
	50	97.7	89.6	87.5
	75	98.1	86.9	85.2
	100	98.1	82.7	81.2
N_2+O_2-40 Pa	25	97.2	95.5	92.8
	50	97.6	91.0	88.6
	75	98.0	85.5	83.7
	100	98.5	81.4	80.1
O_2:N_2-16 Pa	25	98.3	96.3	94.7
	50	97.9	93.0	91.0
	75	98.1	89.5	87.8
	100	98.1	86.5	84.9
O_2:N_2-4 Pa	25	94.6	94.3	89.2
	50	96.7	90.1	87.1
	75	96.8	86.4	83.6
	100	97.5	83.2	81.1

Characterization of the WJM-produced graphene flakes

The morphology of the graphene flakes produced through WJM method was evaluated by TEM) (**Figure** AFM. The TEM image of representative fakes shows that they have an irregular shape with straight borders (**Figure S9a**). The upper right inset shows the electron diffraction pattern corresponding to the TEM image, demonstrating the crystalline nature of the flakes. **Figure S9b** shows an AFM image of the graphene flakes, which have a nanometer thickness. More quantitatively, the statistical analysis of the TEM lateral size (**Figure S8c**) and the AFM thickness (**Figure S9d**) of the flakes show that the data approximately follow lognormal distributions with peaks at ~460 nm and ~1.6 nm, respectively. Therefore, WJM-produced graphene mainly consists of single-/few-layer graphene (SLG/FLG).

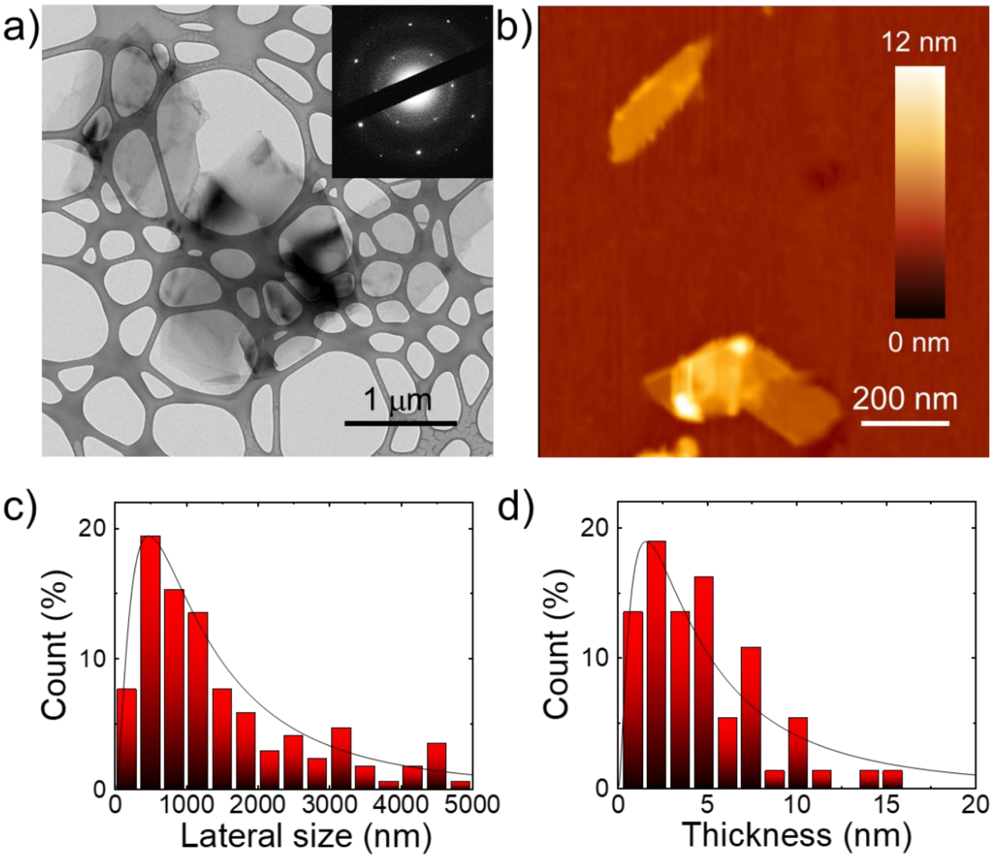

Figure S9. a) Representative TEM image and b) AFM image of SLG/FLG. The upper right inset in panel (a) shows the electron diffraction pattern of the TEM image. (c) TEM statistical analysis of the lateral dimension of the SLG/FLG (acquired on 170 flakes). d) AFM statistical analysis of the thickness of the SLG/FLG (acquired on 100 flakes).

The structural properties of the as-produced flakes were evaluated by Raman spectroscopy, as shown in abundant literature.[11,12,13,14,15,16,16] In general, the Raman spectrum of graphene shows, as fingerprints, G (~1585 cm^{-1}), D (~1380 cm^{-1}), D' (~1620 cm^{-1}) and 2D (~2700 cm^{-1}) peaks[11,12,13,16,17,18]. The G peak, positioned at ~1585 cm^{-1}, corresponds to the E_{2g} phonon at the Brillouin zone centre.[12,11] The D peak is due to the breathing modes of sp^2 rings and requires a defect for its activation by double resonance.[19,20,21] Double resonance happens as an intra-valley process, *i.e.,* connecting two points belonging to the same cone around K or K',[12,11,13] giving origin to the D' peak.[12,11, 13] The 2D peak is the second order of the D peak, [12,11,13] and it appears also in the absence of D peak, since no defects are required for the activation of two phonons with the same momentum, one backscattered from the other.[22,14] Importantly, the thickness of the graphene flakes can be estimated by the analysis of the 2D peak.[16,23] In fact, graphite exhibits a 2D peak that results from two contributions, named $2D_1$ and $2D_2$. The intensity of the $2D_2$ is around twice that of $2D_1$.[12,16,24] Multi-layer graphene (> 5

layers) exhibits a 2D peak, which is almost identical, in term of intensity and lineshape, to the graphite case.[16,25,26] Few-layer graphene, instead, has a $2D_1$ peak more intense than the $2D_2$.[12,16,24] Lastly, SLG exhibits a 2D peak given by a single sharp contribution, which corresponds to $2D_1$.[11,12,13,16] Thus, in the $I(2D_1)/I(G)$ *vs.* $I(2D_2)/I(G)$ plot, the data that fall above that line ($I(2D_2) > I(2D_1)$) correspond to flakes with less than 5 layers, while those below ($I(2D_2) < I(2D_1)$) refer to flakes with more than 5 layers (indistinguishable from graphite). **Figure S10a** shows the Raman spectra of the starting graphite and the WJM-processed sample, normalized to the G peak. The Raman spectrum of the WJM-processed sample exhibited an increase of D peak, compared to those of native graphite., in agreement with previous studies on graphene flakes and graphite.[25,27,28,29,30] These studies have shown that intrinsic defects are located at the edges of graphene flakes and, in absence of defective basal plane, I(D)/I(G) varies inversely with the crystal size.[25,27,28,29,30] However, defects can also occur on the basal planes of the flakes. For such flakes, the I(D)/I(G) correlates with the amount of disorder.[25,27,28,29,30] In order to assess the crystal quality of the graphene flakes, the plot of I(D)/I(G) *vs*. FWHM(G) can be used to identify the presence and nature of defects.[31,32] The plot of I(D)/I(G) *vs*. FWHM(G) (**Figure S10b**) does not show a linear correlation, which means that WJM process does not induce in-plane defects of the sample.[31,32] Lastly, $I(2D_1)/I(G)$ *vs.* $I(2D_2)/I(G)$ plot analysis (**Figure S10c**) indicates that the WJM-processed sample mainly consists of SLG/FLG.

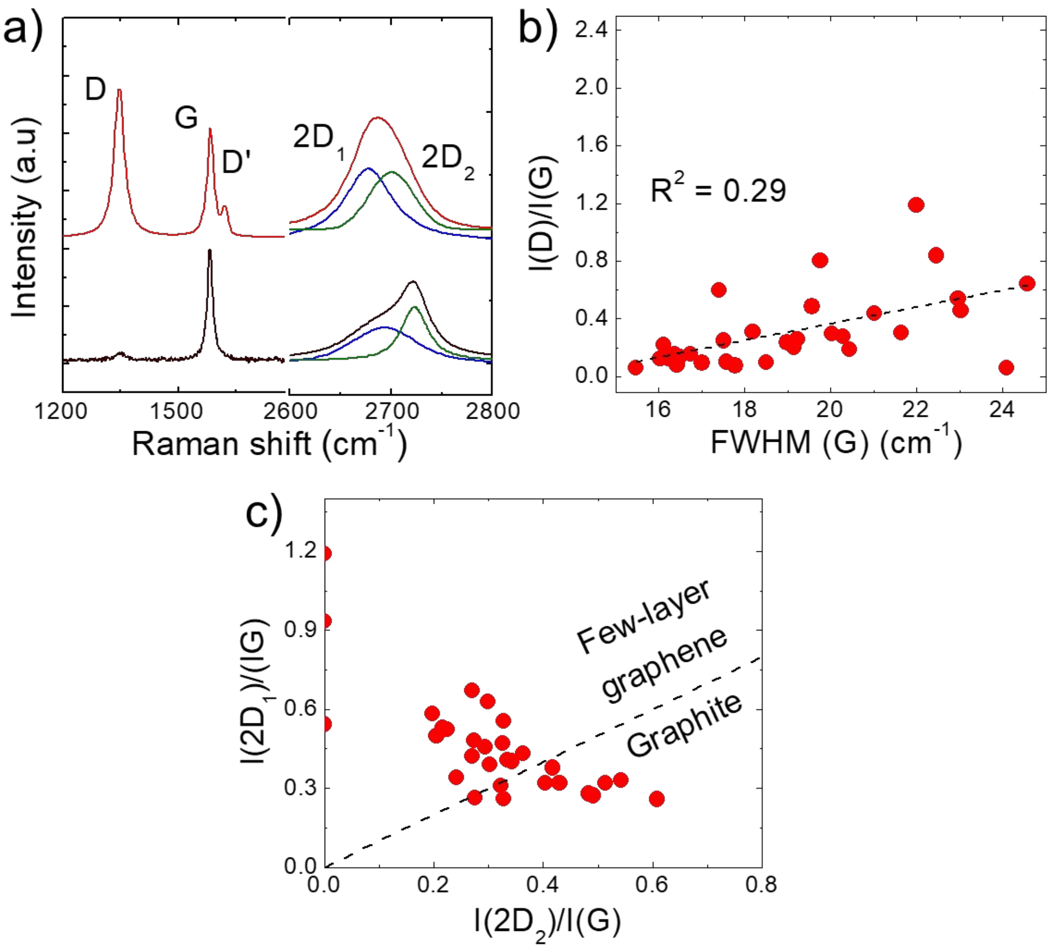

Figure S10. a) Comparison between the Raman spectra (normalized on G peak) of the graphite (black) and WJM-produced graphene flakes (red), with their multi-peak Lorentzian fitting showing the contribution of the individual modes (blue line: $2D_1$; green line: $2D_2$). b) I(D)/I(G) *vs*. FWHM(G) plot for the WJM-produced graphene flakes. c) $I(2D_1)/I(G)$ *vs*. $I(2D_2)/I(G)$ plot for the WJM-produced graphene flakes. The dashed line $I(2D_1)/I(G) = I(2D_2)/I(G)$ represents the multilayer condition (~5 layers).

Polarization curve analysis for the VRFBs based on plasma-treated graphene-decorated GFs

Figure S11a shows the iR-corrected and the raw polarization curves (without iR correction) measured for the VRFBs based on combined plasma-treated graphene-decorated GFs (*i.e.*, graphene-O_2:N_2-16 Pa and graphene-O_2:N_2-4 Pa), in comparison to the curve measured for the reference without graphene (*i.e.*, O_2:N_2-16 Pa). The incorporation of graphene flakes into the GF reduces the kinetic losses of the reference cell. The comparison of the iR-corrected and raw curves **(Figure S11b)** indicates that the Nafion 115 resistance significantly contributes to the overall polarization at current densities higher than 50 mA cm^{-2}.

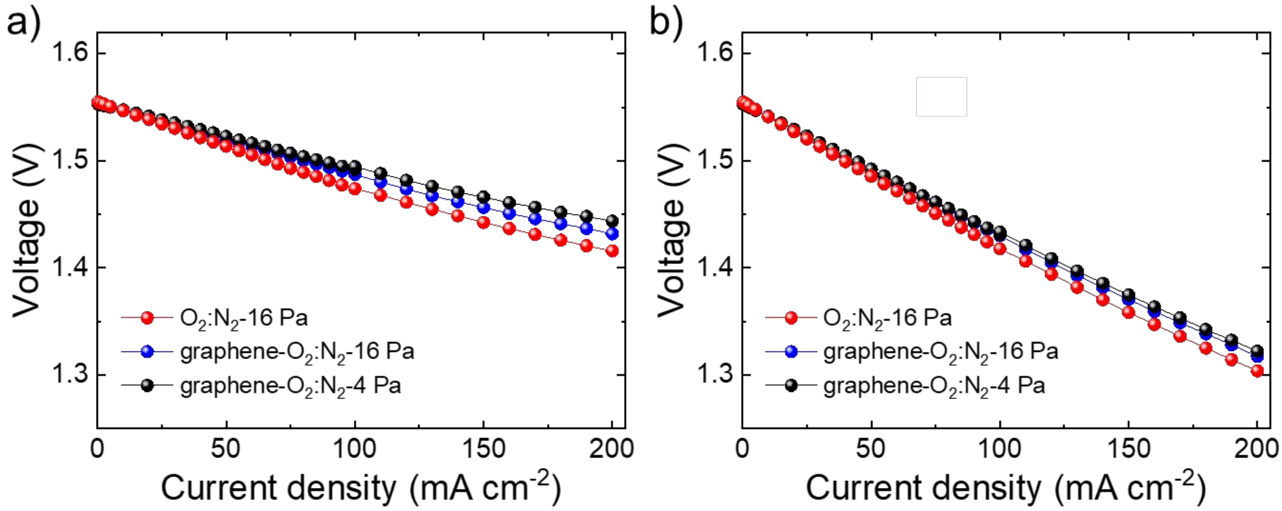

Figure S11. a) iR-corrected polarization curves and b) raw polarization curves (without iR-correction) measured for the VRFBs using plasma-treated graphene-decorated GFs (*i.e.*, graphene-O_2:N_2-16 Pa and graphene-O_2:N_2-4 Pa) and Nafion 115 as the proton-exchange membrane. The polarization curves measured for the optimized graphene-free VRFBs (*i.e.*, O_2:N_2-16 Pa) are also shown.

Galvanostatic CD curves of VRFBs based on plasma-treated electrodes with and without graphene

Figure S12 shows the comparison between the CD curves (second cycle) measured for the optimized VRFBs with and without graphene (*i.e.*, graphene-O_2:N_2-4 Pa and O_2:N_2-16 Pa, respectively) at the current density of 100 mA cm^{-2}. The graphene-O_2:N_2-4 Pa VRFB exhibits the highest discharge capacity of 11.9 Ah L^{-1}, which corresponds to an EU of 88.8% (+6.2% compared to the graphene-free reference).

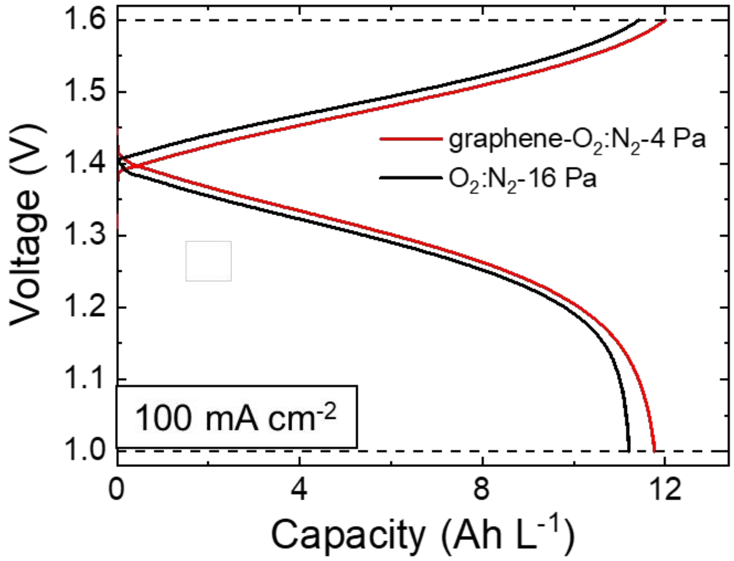

Figure S12. Comparison between the CD curves measured for the optimized VRFBs based on plasma-treated electrodes with and without graphene (*i.e.*, graphene-O_2:N_2-4 Pa and O_2:N_2-16 Pa, respectively).

Polarization curve analysis for the graphene-O_2:N_2-4 Pa VRFBs using Nafion 115 or Nafion XL

Figure S13 shows the polarization curves (with and without iR-correction) measured for graphene-O_2:N_2-4 Pa VRFBs using Nafion 115 or Nafion XL. The raw data indicate that the impact of the ohmic polarization losses are significantly weakened for the case of Nafion XL, while all the other polarization losses attributed to the electrodes are unchanged, being the electrodes the same in the VRFBs.

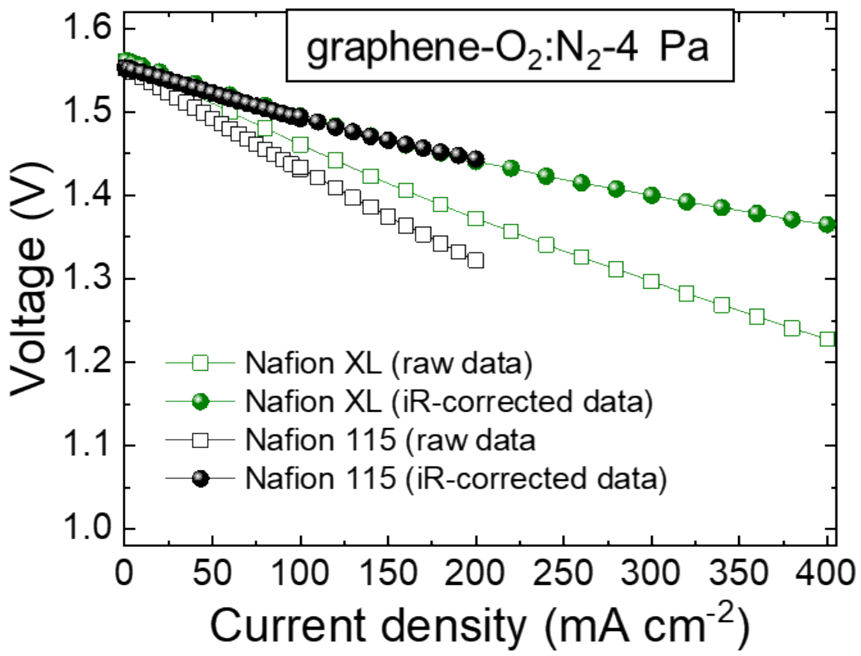

Figure S13. a) iR-corrected polarization curves and b) raw polarization curves (without iR-correction) measured for the VRFBs using plasma-treated graphene-decorated GFs (*i.e.*, graphene-O_2:N_2-16 Pa and graphene-O_2:N_2-4 Pa) and Nafion 115 as the proton-exchange membrane.

References

(1) Del Rio Castillo, A. E.; Pellegrini, V.; Ansaldo, A.; Ricciardella, F.; Sun, H.; Marasco, L.; Buha, J.; Dang, Z.; Gagliani, L.; Lago, E.; Curreli, N.; Gentiluomo, S.; Palazon, F.; Prato, M.; Oropesa-Nuñez, R.; Toth, P. S.; Mantero, E.; Crugliano, M.; Gamucci, A.; Tomadin, A.; Polini, M.; Bonaccorso, F. High-Yield Production of 2D Crystals by Wet-Jet Milling. *Mater. Horizons* **2018**, *5* (5), 890–904.

(2) Del Rio-Castillo, A. E.; Ansaldo, A.; Pellegrini, V.; Bonaccorso, F. Exfoliation Materials by Wet-Jet Milling Techniques. WO2017/089987A1, 2017.

(3) Chen, J.-Z.; Liao, W.-Y.; Hsieh, W.-Y.; Hsu, C.-C.; Chen, Y.-S. All-Vanadium Redox Flow Batteries with Graphite Felt Electrodes Treated by Atmospheric Pressure Plasma Jets. *J. Power Sources* **2015**, *274*, 894–898.

(4) Estevez, L.; Reed, D.; Nie, Z.; Schwarz, A. M.; Nandasiri, M. I.; Kizewski, J. P.; Wang, W.; Thomsen, E.; Liu, J.; Zhang, J.-G.; Sprenkle, V.; Li, B. Tunable Oxygen Functional Groups as Electrocatalysts on Graphite Felt Surfaces for All-Vanadium Flow Batteries. *ChemSusChem* **2016**, *9* (12), 1455–1461.

(5) Deng, Q.; Huang, P.; Zhou, W.-X.; Ma, Q.; Zhou, N.; Xie, H.; Ling, W.; Zhou, C.-J.; Yin, Y.-X.; Wu, X.-W.; Lu, X.-Y.; Guo, Y.-G. A High-Performance Composite Electrode for Vanadium Redox Flow Batteries. *Adv. Energy Mater.* **2017**, *7* (18), 1700461.

(6) Yue, L.; Li, W.; Sun, F.; Zhao, L.; Xing, L. Highly Hydroxylated Carbon Fibres as Electrode Materials of All-Vanadium Redox Flow Battery. *Carbon N. Y.* **2010**, *48* (11), 3079–3090.

(7) Blume, R.; Rosenthal, D.; Tessonnier, J.-P.; Li, H.; Knop-Gericke, A.; Schlögl, R. Characterizing Graphitic Carbon with X-Ray Photoelectron Spectroscopy: A Step-by-Step Approach. *ChemCatChem* **2015**, *7* (18), 2871–2881.

(8) Kumar, N. A.; Nolan, H.; McEvoy, N.; Rezvani, E.; Doyle, R. L.; Lyons, M. E. G.; Duesberg, G. S. Plasma-Assisted Simultaneous Reduction and Nitrogen Doping of Graphene Oxide Nanosheets. *J. Mater. Chem. A* **2013**, *1* (14), 4431.

(9) Gonella, G.; Terreni, S.; Cvetko, D.; Cossaro, A.; Mattera, L.; Cavalleri, O.; Rolandi, R.; Morgante, A.; Floreano, L.; Canepa, M. Ultrahigh Vacuum Deposition of <scp>l</Scp> -Cysteine on Au(110) Studied by High-Resolution X-Ray Photoemission: From Early Stages of Adsorption to Molecular Organization. *J. Phys. Chem. B* **2005**, *109* (38), 18003–18009.

(10) Negro, E.; Nale, A.; Vezzù, K.; Pagot, G.; Polizzi, S.; Bertoncello, R.; Ansaldo, A.; Prato, M.; Bonaccorso, F.; Rutkowska, I. A.; Kulesza, P. J.; Di Noto, V. Hierarchical Oxygen Reduction Reaction Electrocatalysts Based on FeSn0.5 Species Embedded in Carbon Nitride-Graphene Based Supports. *Electrochim. Acta* **2018**, *280*, 149–162.

(11) Ferrari, A. C.; Meyer, J. C.; Scardaci, V.; Casiraghi, C.; Lazzeri, M.; Mauri, F.; Piscanec, S.; Jiang, D.; Novoselov, K. S.; Roth, S.; Geim, A. K. Raman Spectrum of Graphene and Graphene Layers. *Phys. Rev. Lett.* **2006**, *97* (18), 187401.

(12) Ferrari, A. C.; Basko, D. M. Raman Spectroscopy as a Versatile Tool for Studying the Properties of Graphene. *Nature Nanotechnology*. Nature Publishing Group, a division of Macmillan Publishers Limited. All Rights Reserved. April 4, 2013, pp 235–246.

(13) Najafi, L.; Bellani, S.; Martín-García, B.; Oropesa-Nunez, R.; Del Rio Castillo, A. E.; Prato, M.; Moreels, I.; Bonaccorso, F. Solution-Processed Hybrid Graphene Flake/2H-MoS2 Quantum Dot Heterostructures for Efficient Electrochemical Hydrogen Evolution. *Chem. Mater.* **2017**, *29* (14), 5782–

5786.
(14) Bellani, S.; Najafi, L.; Martín-García, B.; Ansaldo, A.; Del Rio Castillo, A. E.; Prato, M.; Moreels, I.; Bonaccorso, F. Graphene-Based Hole-Selective Layers for High-Efficiency, Solution-Processed, Large-Area, Flexible, Hydrogen-Evolving Organic Photocathodes. *J. Phys. Chem. C* **2017**, *121* (40), 21887–21903.
(15) Dresselhaus, M. S.; Jorio, A.; Hofmann, M.; Dresselhaus, G.; Saito, R. Perspectives on Carbon Nanotubes and Graphene Raman Spectroscopy. *Nano Lett.* **2010**, *10* (3), 751–758.
(16) Wu, J.-B.; Lin, M.-L.; Cong, X.; Liu, H.-N.; Tan, P.-H. Raman Spectroscopy of Graphene-Based Materials and Its Applications in Related Devices. *Chem. Soc. Rev.* **2018**, *47* (5), 1822–1873.
(17) Su, C.-Y.; Xu, Y.; Zhang, W.; Zhao, J.; Tang, X.; Tsai, C.-H.; Li, L.-J. Electrical and Spectroscopic Characterizations of Ultra-Large Reduced Graphene Oxide Monolayers. *Chem. Mater.* **2009**, *21* (23), 5674–5680.
(18) Thomsen, C.; Reich, S. Double Resonant Raman Scattering in Graphite. *Phys. Rev. Lett.* **2000**, *85* (24), 5214–5217.
(19) Yang, L.; Deslippe, J.; Park, C.-H.; Cohen, M. L.; Louie, S. G. Excitonic Effects on the Optical Response of Graphene and Bilayer Graphene. *Phys. Rev. Lett.* **2009**, *103* (18), 186802.
(20) Ferrari, A. C.; Robertson, J. Interpretation of Raman Spectra of Disordered and Amorphous Carbon. *Phys. Rev. B* **2000**, *61* (20), 14095–14107.
(21) Ferrari, A. C.; Robertson, J. Resonant Raman Spectroscopy of Disordered, Amorphous, and Diamondlike Carbon. *Phys. Rev. B* **2001**, *64* (7), 075414.
(22) Su, C.-Y.; Xu, Y.; Zhang, W.; Zhao, J.; Tang, X.; Tsai, C.-H.; Li, L.-J. Electrical and Spectroscopic Characterizations of Ultra-Large Reduced Graphene Oxide Monolayers. *Chem. Mater.* **2009**, *21* (23), 5674–5680.
(23) Nuvoli, D.; Valentini, L.; Alzari, V.; Scognamillo, S.; Bon, S. B.; Piccinini, M.; Illescas, J.; Mariani, A. High Concentration Few-Layer Graphene Sheets Obtained by Liquid Phase Exfoliation of Graphite in Ionic Liquid. *J. Mater. Chem.* **2011**, *21* (10), 3428–3431.
(24) Dresselhaus, M. .; Dresselhaus, G.; Hofmann, M. Raman Spectroscopy as a Probe of Graphene and Carbon Nanotubes. *Philos. Trans. R. Soc. A Math. Phys. Eng. Sci.* **2008**, *366* (1863), 231–236.
(25) Lucchese, M. M.; Stavale, F.; Ferreira, E. H. M.; Vilani, C.; Moutinho, M. V. O.; Capaz, R. B.; Achete, C. A.; Jorio, A. Quantifying Ion-Induced Defects and Raman Relaxation Length in Graphene. *Carbon N. Y.* **2010**, *48* (5), 1592–1597.
(26) Das, A.; Chakraborty, B.; Sood, A. K. Raman Spectroscopy of Graphene on Different Substrates and Influence of Defects. *Bull. Mater. Sci.* **2008**, *31* (3), 579–584.
(27) Eckmann, A.; Felten, A.; Mishchenko, A.; Britnell, L.; Krupke, R.; Novoselov, K. S.; Casiraghi, C. Probing the Nature of Defects in Graphene by Raman Spectroscopy. *Nano Lett.* **2012**, *12* (8), 3925–3930.
(28) Ferrari, A. C. Raman Spectroscopy of Graphene and Graphite: Disorder, Electron–Phonon Coupling, Doping and Nonadiabatic Effects. *Solid State Commun.* **2007**, *143* (1–2), 47–57.
(29) Lotya, M.; Hernandez, Y.; King, P. J.; Smith, R. J.; Nicolosi, V.; Karlsson, L. S.; Blighe, F. M.; De, S.; Wang, Z.; McGovern, I. T.; Duesberg, G. S.; Coleman, J. N. Liquid Phase Production of Graphene by Exfoliation of Graphite in Surfactant/Water Solutions. *J. Am. Chem. Soc.* **2009**, *131* (10), 3611–3620.

(30) Cançado, L. G.; Jorio, A.; Ferreira, E. H. M.; Stavale, F.; Achete, C. A.; Capaz, R. B.; Moutinho, M. V. O.; Lombardo, A.; Kulmala, T. S.; Ferrari, A. C. Quantifying Defects in Graphene via Raman Spectroscopy at Different Excitation Energies. *Nano Lett.* **2011**, *11* (8), 3190–3196.

(31) Bracamonte, M. V.; Lacconi, G. I.; Urreta, S. E.; Foa Torres, L. E. F. On the Nature of Defects in Liquid-Phase Exfoliated Graphene. *J. Phys. Chem. C* **2014**, *118* (28), 15455–15459.

(32) Coleman, J. N. Liquid Exfoliation of Defect-Free Graphene. *Acc. Chem. Res.* **2013**, *46* (1), 14–22.
